# Probing the genomic landscape of human sexuality: a critical systematic review of the literature

**DOI:** 10.3389/fgene.2023.1184758

**Published:** 2023-08-24

**Authors:** Nicola Luigi Bragazzi, Manlio Converti, Andrea Crapanzano, Riccardo Zerbetto, Anna Siri, Rola Khamisy-Farah

**Affiliations:** ^1^ Laboratory for Industrial and Applied Mathematics (LIAM), Department of Mathematics and Statistics, York University, Toronto, ON, Canada; ^2^ Postgraduate School of Public Health, Department of Health Sciences (DISSAL), University of Genoa, Genoa, Italy; ^3^ United Nations Educational, Scientific and Cultural Organization (UNESCO) Chair, Health Anthropology Biosphere and Healing Systems, University of Genoa, Genoa, Italy; ^4^ ASL Napoli 2 Nord, Naples, Italy; ^5^ Department of Counseling, San Francisco State University, San Francisco, CA, United States; ^6^ GESTALT Study Center (CSTG), Milano, Italy; ^7^ Clalit Health Services, Akko, Israel; ^8^ Azrieli Faculty of Medicine, Bar-Ilan University, Safed, Israel

**Keywords:** human sexuality, sexual orientation, behavioral genomics, linkage study, candidate gene study, genome-wide association study (GWAS), critical systematic review

## Abstract

Whether human sexuality is the result of nature or nurture (or their complex interplay) represents a hot, often ideologically driven, and highly polarized debate with political and social ramifications, and with varying, conflicting findings reported in the literature. A number of heritability and behavioral genetics studies, including pedigree-based investigations, have hypothesized inheritance patterns of human sexual behaviors. On the other hand, in most twin, adoption, and nuclear family studies, it was not possible to disentangle between underlying genetic and shared environmental sources. Furthermore, these studies were not able to estimate the precise extent of genetic loading and to shed light both on the number and nature of the putative inherited factors, which remained largely unknown. Molecular genetic studies offer an unprecedented opportunity to overcome these drawbacks, by dissecting the molecular basis of human sexuality and allowing a better understanding of its biological roots if any. However, there exists no systematic review of the molecular genetics of human sexuality. Therefore, we undertook this critical systematic review and appraisal of the literature, with the ambitious aims of filling in these gaps of knowledge, especially from the methodological standpoint, and providing guidance to future studies. Sixteen studies were finally retained and overviewed in the present systematic review study. Seven studies were linkage studies, four studies utilized the candidate gene approach, and five studies were GWAS investigations. Limitations of these studies and implications for further research are discussed.

## Introduction

Whether human sexuality is the result of nature or nurture (or both and of their complex, non-linear interplay) represents a hot, often ideologically driven, highly polarized debate with political and social ramifications (Jannini et al., 2010; Burri et al., 2011; Jannini et al., 2015; Bailey et al., 2016; Bogaert and Skorska, 2020), and with varying, conflicting findings reported in the existing scholarly literature (Eckert et al., 1986).

Sexuality is a multi-dimensional construct that, according to the “World Health Organization” (WHO), can be defined as “a central aspect of being human throughout life”, encompassing “sex, gender identities and roles, sexual orientation, eroticism, pleasure, intimacy, and reproduction. Sexuality is experienced and expressed in thoughts, fantasies, desires, beliefs, attitudes, values, behaviors, practices, roles, and relationships. While sexuality can include all of these dimensions, not all of them are always experienced or expressed. Sexuality is influenced by the interaction of biological, psychological, social, economic, political, cultural, legal, historical, religious, and spiritual factors”. Of note, sexuality includes several aspects, like sex at birth, gender identity, and expression, as well as sexual identity, sexual orientation, sexual activity/sexual behavior, and sexual role, but these, if known/disclosed, cannot be used as proxies to infer the other dimensions, if unknown.

An increasing number of genetic-epidemiological surveys, including heritability and behavioral genetics studies, such as family and pedigree-based investigations (Jannini et al., 2010; Burri et al., 2011; Jannini et al., 2015; Bogaert and Skorska, 2020), have hypothesized inheritance patterns of human sexual behaviors, depicting the familial aggregation and clustering of sexual orientation, its development, and differentiation. They computed a higher frequency of homosexual brothers of homosexual index subjects with respect to heterosexual index subjects. Concurring evidence was provided by twin studies showing higher rates of same-sex sexual behaviors among monozygotic and, to a lesser degree, dizygotic twins (Jannini et al., 2015), compared to ordinary siblings, and adoptive (“adopted in”) brothers and sisters of homosexual individuals, with estimates of heritability in the range of 31%–74% for males and 27%–76% for females (Eckert et al., 1986; Jannini et al., 2010; Burri et al., 2011; Jannini et al., 2015; Bailey et al., 2016; Bogaert and Skorska, 2020).

On the other hand, some investigations reported contrasting findings, with similar rates of sexual behavior in biological and adoptive siblings of male homosexual index subjects (Bailey and Pillard, 1991). Furthermore, in most twin, adoption, and nuclear family studies, it was not possible to disentangle between underlying genetic and shared environmental sources (Eckert et al., 1986). Moreover, these studies were not able to estimate the precise extent of genetic loading and to shed light both on the number and nature of the putative inherited factors, which remain largely unknown.

Molecular genetics and genomic studies offer an unprecedented opportunity to overcome these drawbacks, by dissecting the molecular basis of human sexuality and allowing a better understanding of its biological roots if any (Pillard and Weinrich, 1986; Bailey and Pillard, 1991; Bailey and Benishay, 1993; Pillard and Bailey, 1998; Bailey et al., 1999). However, given the sensitive nature of the topic and the complexity of behavioral genomics, to the best of our knowledge, there exists no systematic review of the molecular genetics and genomics of human sexuality. Therefore, we undertook this critical systematic review and appraisal of the literature, with the ambitious aims of filling in these gaps of knowledge, especially from the methodological standpoint, and providing guidance to future studies exploring the biological landscape of human sexuality.

## Materials and methods

### Study protocol

An *a priori* study protocol was written after preliminary familiarization with the scholarly literature, and can be requested by contacting the corresponding author.

### Research aims and research questions

The research aims were to better understand the genetic basis of human sexuality and sexual orientation, also appraising the methodological quality of published research. The research questions were as follows: “Which are the genetics/genomics basis of human sexuality? To which extent do they contribute to shaping human sexuality? How replicable are these putative genetic/genomic factors?”

### Search strategy

We utilized the following search string: (“human sexuality” OR “sexual identity” OR “sexual orientation” OR “same-sex behavior” OR “same-sex sexual behavior” OR “same-sex desire” OR “same-sex attraction” OR “same-sex phantasy” OR “same-gender sexuality” OR “sexual typicality” OR “gender typicality” OR “gender nonconformity” OR “gender incongruence” OR “gender identity” OR “gender expression” OR “gender diverse” OR “gender variant” OR “sexual fluidity” OR “sexual plasticity” OR “sexual diversity” OR “sexual minority” OR “sexual and gender minorities” OR homosexual OR homosexuality OR bisexual OR bisexuality OR asexual OR asexuality OR lesbian OR pansexual OR pansexuality OR intersex OR intersexuality OR transgender OR transman OR transmen OR transwoman OR transwomen OR female-to-male OR male-to-female OR 2SLGBTQI + OR LGBT OR LGBT + OR LGBTQ OR LGBTQ + OR LGBTQI + OR “men having sex with men” OR “men who have sex with men” OR “MSM community” OR “MSM population”) AND (gene OR genome OR genetic OR “genetic component” OR “genetic factor” OR “genetic association” OR “Genome-Wide Linkage” OR GWL OR “DNA marker” OR “genome-wide association study” OR GWAS OR “genetic locus” OR “genetic loci” OR “genome-wide scan” OR “genetic architecture” OR “genetic landscape” OR “genetic influence” OR “genomic architecture” OR “genomic landscape” OR “microsatellite marker” OR polymorphism OR SNP OR “single nucleotide polymorphism” OR “genetic variant” OR “genetic variation” OR “DNA variant” OR “DNA variation” OR “linkage disequilibrium” OR zygosity OR homozygosity OR heterozygosity OR allele OR allelic OR haplotype OR “genome-wide association meta-analyses” OR GWAMA OR “candidate gene study” OR “transcriptome-wide association studies” OR TWAS OR transcriptome OR transcriptomics) NOT (“animal study” OR “animal model” OR “*in vitro*”). This search string was elaborated based on three major pre-identified domains: namely, 1) human sexuality, 2) genetic/genomic aspects, and 3) human subjects/populations. The search string was developed with the help of a librarian and was revised and refined after an initial familiarization with the literature.

### Inclusion and exclusion criteria

MEDLINE, a major electronic, scholarly database, consisting of more than 35 million records from the biomedical literature, was mined via its freely available interface (PubMed) from its inception, without time or language filters. Inclusion/exclusion criteria were devised according to the “Sample, Phenomenon of Interest, Design, Evaluation, Research type” (SPIDER) acronym (Methley et al., 2014). Specifically, we looked for genetic epidemiological studies employing molecular genetic techniques, and in particular, genome-wide linkage studies (GWL), which look at the relation between the transmission of a given *locus* and the disease/trait of interest within families, large-scale genome-wide association studies (GWAS), which focus their analysis on the relation between a specific allele and the disease/trait within population, genome-wide association meta-analyses (GWAMA), and transcriptome-wide association studies (TWAS), attempting to isolate and identify chromosomal and genetic regions and their transcripts relevant to sexual orientation.

Besides genetic linkage and association studies in families/populations, candidate gene studies were also considered. We discarded heritability and behavioral genetics studies; in that they are unable 1) to disentangle between biological (genetic) and environmental/socio-cultural factors and 2) estimate the contribution of every single variable and their interactions to shaping complex behaviors and traits.

Studies focusing on human populations and investigating members of the two spirit-lesbian-gay-bisexual-transsexual/transgender-queer-intersex-asexual-polysexual (2SLGBTQIAP+) community were included with the exception of those focusing on the disorders of sex development (DSD), whilst bioinformatic analyses, animal models, or *in vitro* studies were not retained. They were considered only if conducted as follow-up studies of genetic-epidemiological investigations, to provide more biological insights into the genetic *loci* identified/discovered.

Any study design/publication type (original research article, correspondence with original data, follow-up study/replication study, or meta-analytical study) was deemed eligible for inclusion, whilst editorials, letters to the editor without original data, commentaries, or technical notes merely focusing on genetic/genomic methodologies were excluded. Extant review studies, if any, were scanned to ensure that relevant articles were not missed but were not retained in the present systematic review of the literature.

Extensive cross-referencing was applied, to ensure broad and relevant coverage of the topic. Target journals, like the “American Journal of Human Genetics,” “Archives of Sexual Behavior,” “Hormones and Behavior,” “Journal of Andrology,” “Journal of Sexual Medicine,” “Journal of Sex Research,” and “Sexual Medicine Reviews,” were hand-searched. Finally, findings were reported according to the “Preferred Reporting Items for Systematic reviews and Meta-Analyses” (PRISMA) guidelines (Page et al., 2021).

## Results

The initial literature search yielded a pool of 32,404 items. After removing irrelevant items, using an Artificial intelligence-enhanced tool based on a text mining algorithm, twenty-six studies were assessed in full-text. Out of these 26 studies, the following ten studies were excluded with reason: the studies by Cantor et al. (2002), Iemmola and Camperio Ciani (2009), Hamer (1999), Rice et al. (1995), Rice et al. (1999b), Sanders et al. (1998), Sanders and Dawood (2003), Zietsch et al. (2021), Hamer et al. (2021), and Ganna et al. (2021). More specifically, (Cantor et al., 2002; Iemmola and Camperio Ciani, 2009), were excluded since they are formal genetic studies, without molecular insights, (Hamer, 1999; Rice et al.1999b; Hamer et al., 2021; Ganna et al., 2021), were letters to editor/replies or technical comments without sufficient quantitative details, (Rice et al., 1995), was a conference presentation, (Sanders and Dawood, 2003), was a non-peer-reviewed publication, and (Zietsch et al., 2021) was a computational simulation, without molecular insights.

Sixteen studies were finally retained and overviewed in the present systematic review study. Seven studies (Hamer et al., 1993; Hu et al., 1995; Rice et al., 1999a; Mustanski et al., 2005; Ramagopalan et al., 2010; Sanders et al., 2015; Sanders et al., 2021a) were linkage studies, four studies (Macke et al., 1993; DuPree et al., 2004; Yu et al., 2015; Qin et al., 2018) utilized the candidate gene approach, and five studies (Wang et al., 2012; Lawrance-Owen et al., 2013; Sanders et al., 2017; Ganna et al., 2019; Hu et al., 2021) were GWAS investigations.

### Linkage studies of human sexual orientation

In 1993, Hamer and colleagues (Hamer et al., 1993) were able to find strong support for a genetic component in male sexual orientation, based on the observation of family recurrence patterns and molecular analysis of the X chromosome in sibships characterized by multiple homosexual brothers. More specifically, the authors were the first to suggest “that a *locus* (or *loci*) related to sexual orientation lies within approximately 4 million base pairs of DNA on the tip of the long arm of the X chromosome”. The authors were able to exploit the development of chromosomal genetic maps densely populated with highly polymorphic markers, which enabled the application of the standard techniques of modem human genetics (pedigree analysis and family DNA linkage studies) to the study of complex phenotypes and traits, including human sexuality. In this study, homosexuality was operationalized as scoring 5 or 6 on the “Heterosexual-Homosexual Rating Scale”, commonly referred to as the “Kinsey Scale”, homosexual individuals were also individuals acknowledged as such to the proband or another family member, whilst in all the other cases individuals were categorized as non-homosexual (heterosexual, bisexual, or of unclear sexual orientation). Seventy-six gay male index subjects were recruited through outpatient “Human Immunodeficiency Virus” (HIV) clinics and homophilic organizations: these constituted the randomly ascertained pool of the study. One or more relatives from 26 of these families were also included, whilst the sample for the sib-pair pedigree study comprised 38 pairs of homosexual brothers, along with their parents or other relatives, recruited through advertisements in homophilic publications. Finally, two additional families from the randomly ascertained pool were retained in the DNA linkage study. In total, the investigators analyzed 114 families of homosexual men (92% white non-Hispanic, 4% African American, 3% Hispanic, and 1% Asian, with an average age of 36 ± 9 years and an average educational level of 15.5 ± 2.4 years), finding increased rates of same-sex orientation in their maternal uncles and male cousins through maternal aunts, but not in their fathers or paternal relatives. As such, the authors hypothesized a sex-linked transmission of homosexuality at least in a portion of the population: then, the analysis was restricted to a selected group of 40 families with two gay brothers and without non-maternal transmission, leading to the discovery of statistically significant correlations between homosexuality and the inheritance of polymorphic markers located on the X chromosome in about 64% of the sib-pairs tested. The genetic linkage to markers on Xq28, the subtelomeric region of the long arm of the sex chromosome, yielded a multipoint LOD score of 4.0 (*p*-value of 10^–5^), indicating, with a high statistical confidence level (>99%), that, at least, one (sub-) type of male sexual orientation can be inherited and is genetically influenced. Of note, the population studied was relatively young and exhibited bimodal distributions of Kinsey scores. This study was criticized (Rice et al., 1999a) for several methodological deficiencies, including the lack of DNA linkage analysis in the population consisting of non-gay siblings of the homosexual subjects analyzed. The absence of a control group represents, indeed, a serious shortcoming of the investigation. Moreover, given that male homosexual orientation and sexuality, in general, are not simple Mendelian traits, but, rather, complex phenotypes, the alleged Hamer’s gene contributing to male homosexuality would be subjected to strong selective pressure, even though the so-called “sexually antagonistic hypothesis” exactly predicts human sexuality-related *loci* to be highly over-represented on chromosome X, with up to a couple of *loci* there, linking sexual attraction and reproduction, and allowing for strong asymmetries, with large decreases in reproductive fitness in males being counterbalanced by a relatively small increase in females. Moreover, the “sexually antagonistic hypothesis” would explain sex- and gender-specific differences in psychology (i.e., personality traits) and health-related outcomes. Furthermore, the Hamer’s report would suffer from type 1 (false positive) error, making it, as such, irreproducible. Finally, scoring of allele sharing was not performed in a blinded way and different laboratory steps (phenotypic characterizations and genotypic analyses) were conducted by the same person(s).

In a follow-up study conducted in 1995 (Hu et al., 1995), the authors extended their research to two newly ascertained series of families that contained either two gay brothers or two lesbian sisters as well as heterosexual siblings. Out of the 33 gay male sibling pairs (32 of which informative), 22 shared all the Xq28 markers, corroborating the previous findings of a reported linkage between Xq28 and male homosexuality in selected kinships. The authors added that this region may contain a *locus* that contributes to shaping individual variations in sexual orientation in men but not in women.

However, these findings (Hamer et al., 1993; Hu et al., 1995) were challenged by Rice et al. (1999a), who tried, in 1999, but failed to replicate the Xq28 linkage. More in detail, sharing of alleles at position Xq28 was investigated in a sample recruited from 182 Canadian families. The sampling technique adopted was similar to that employed by Hamer et al. (1993) (Hu et al., 1995): the Canadian scholars advertised their study protocol in local gay news magazines, specifically looking for families with high rates of sibling concordance in terms of sexual orientation (at least two gay brothers). The families recruited included 614 brothers, 269 (44%) of whom were homosexual. There were 148 families with two gay sons, 34 families with three, and two families with four. The sample included 270 sisters, 49 (18%) of whom were homosexual, a rate higher than the frequency found in most population-based studies, which suggest a sister concordance rate of 14%. The molecular analysis was applied to a sub-sample consisting of 52 gay male sibling pairs from 48 families (46 families with two gay brothers and two families with three gay brothers, “six pairs”, or “two sib trios”), with 33 additional sibling pairs concordant for multiple sclerosis acting as controls. In this study, homosexuality was operationalized as replying yes to a direct question asked by a gay interviewer. Moreover, the index subject had to confirm if he defined himself as gay and if consumed gay magazines. His gay brother had to corroborate this self-definition. Four markers at Xq28 were assessed (namely, DXS1113, BGN, Factor 8, and DXS1108) along a 12.5-centimorgan (cM) region of Xq28. Allele and haplotype sharing for these markers was not increased over expectation, not supporting an X-linked gene underlying homosexuality in males. More specifically, the sharing of distal Xq28 markers in the 46 sib pairs was 20/46. Concerning the two sib trios, for one of them, all three brothers shared the same X chromosome, whilst, for the other trio, two shared the same X chromosome and the other was different. Overall, 23 out of 50 chromosomes (46%) were shared in the analyzed sample. Also, after excluding and removing families if a father was gay or if there were any first-degree lesbian relatives, the lack of linkage evidence would remain unchanged.

Furthermore, Hamer (Hamer, 1999) replied to Rice et al. (1999a) criticisms, arguing that the Canadian researchers had published analysis conducted on a subset of their initial recruited sample (Rice et al., 1995), not representative of the published dataset. The initial sample consisted of probands from 182 families that had at least two gay brothers, finding that 13.4% (35/261) of their maternal uncles were gay as compared to 6.9% (24/364) of their paternal uncles, pointing to maternal transmission of male homosexuality, exactly as predicted by the Xq28 hypothesis. Reporting bias of male homosexual behavior could be ruled out on the basis of a slight excess of lesbian aunts on the paternal side of the family. Moreover, even though the published study (Rice et al., 1999a) was larger than the Hamer et al. (1993) (Hu et al., 1995) study in terms of sample size, it may be underpowered to capture allele sharing: having a population of 52 sib-pairs, power to detect (at least) 64% allele sharing at the 0.05 level of significance was only 65%, with a 35% chance that linkage may have not been detected simply by chance. Also, criteria for studying male homosexuality were not validated and, probably, were unreliable, being based on a single direct question. Moreover, Hamer (Hamer, 1999) performed a meta-analysis of the previously published and unpublished linkage studies (Hamer et al., 1993; Hu et al., 1995; Sanders et al., 1998; Rice et al., 1999a), computing a pooled level of allele sharing of 64% (*p* = 0.0001), which is statistically significant and corresponds to a λ_s_ value of 1.4, the ratio for male homosexuality in the brothers of a gay index subject, with respect to the population frequency attributable to a gene in this region. All this seems to suggest a modest level of influence, which can be expected for a single *locus* when the trait of interest is complex, confirming the Xq28 hypothesis.

Sanders and Dawood (2003) reported and discussed an unpublished report, finding no support of X-linked heritage of human male homosexuality and performed a combined analysis of the results previously conducted, including theirs: the quantitative synthesis of four studies (Hamer et al., 1993; Hu et al., 1995; Rice et al., 1999a; Sanders and Dawood, 2003) yielded a statistically suggestive multiple scan probability (MSP) value of 0.00003.

However, even though suggestive, the Xq28 hypothesis can account for, at most, only a small portion of the overall heritability of male homosexual orientation as shown by family and twin studies, which showed evidence of non-maternal transmission and, therefore, of the impact of other contributory *loci*. As such, leveraging genomewide linkage scanning technique could help identify genes involved in human sexuality (and, more specifically, sexual orientation), without having to use paternal transmission pattern and, as such, exclude some families from the enrollment.

Subsequently, two major genetic/genomic datasets have been released, which account for the majority (>90%) of the published GWLS concordant sibling pairs on the male homosexual trait: namely, 1) the “Molecular Genetic Study of Sexual Orientation” (MGSOSO), which consists of 409 concordant sibling pairs, 908 analyzed individuals in 384 independent multiplex families (793 homosexual brothers, 33 heterosexual brothers, 49 mothers, and 33 fathers), from several primarily English-speaking countries, such as the United States (98.2%), Canada (1.6%), and the United Kingdom (0.2%), published by Sanders and colleagues in 2015 (Sanders et al., 2015), and 1) the so-called “Hamer dataset”, which comprises 155 concordant sibling pairs, 456 analyzed individuals in 146 unrelated families with two or more gay brothers (73 previously reported families and 73 newly reported families; 137 families with two gay brothers and nine families with three gay brothers; thirty of the families with one parent, 30 of the families with both parents; 46 of the families with at least one heterosexual male or female full sibling - up to 6 additional siblings per family), published by Mustanski et al. (2005).

The former study (Sanders et al., 2015) utilized a participatory research approach, by looking at families with at least two gay brothers and recruiting probands through booths at 2SLGBTQIAP + community festivals (like Gay Pride and related festivals), through advertisements and articles in homophilic media, liaisons with 2SLGBTQIAP + groups, and an educationally oriented internet site. Any other member in a family (parents and brothers) was enrolled, regardless of their sexual orientation, through the proband, by snowballing. Most (97.9%) of the studied families were of European ancestry, while the others self-identified as African American (1.6%) and Asian (0.5%). The large majority (95.1%) were non-Hispanic, whereas 4.9% were Hispanic. The average age of the recruited brothers was 44.3 ± 10.7 years, ranging from 18.7 to 88.9 years. In this study, homosexuality was operationalized as a primarily bimodal psychological trait: homosexuals were defined as individuals of self-reported homosexual identity and past-year Kinsey self-report questionnaire scores 5–6 for feelings, i.e., sexual fantasies, while heterosexuals were individuals of heterosexual identity and past-year Kinsey self-report questionnaire scores 0–1. Out of the 793 analyzed homosexual brothers, 708 (89%) were Kinsey 6, and 85 (11%) Kinsey 5; out of the 33 heterosexual brothers, 31 (94%) were Kinsey 0, and 2 (6%) Kinsey 1. Out of 384 families, 361 had two homosexual brothers, 21 families had three, and two families had four, yielding a total of 409 independent homosexual brother pairs. Collected and analyzed samples were saliva and blood. Genotyping was performed by SNP, while a supplementary chromosome X analysis dataset (consisting of 146 genotyped individuals from 50 families, forming 56 independent homosexual brother pairs) was genotyped by simple tandem repeat polymorphism (STRP). The authors conducted two-point and multipoint analysis for linkage using MERLIN non-parametric linkage employing the Kong-Cox linear model and the S-pairs option to assess independent sibling pairs. Of note, the well-known “fraternal birth order effect”—that is to say, the environmental contribution of more older biological brothers from the same mother increasing the chance later born men would be homosexual—was incorporated into linkage analyses for the two strongest peaks by means of parametric models (dominant, recessive, and X-linked) using variable penetrance estimates to simulate increasing phenocopy rates paralleling the increase in the number of older brothers. Suggestive two-point linkage (LOD≥2.2) could be computed for 352 SNPs, with five of these five exceeding the threshold for genome-wide significance (LOD≥3.6): namely, 1) rs13212974, an intergenic SNP, with the nearest gene being FRK, coding for the fyn-related kinase; 2) rs6990254 at CLVS1 (clavesin 1); 3) rs2498600 at PTPRD (protein tyrosine phosphatase, receptor type, D); 4) rs2221108 at GRM5 (glutamate receptor, metabotropic 5); and 5) rs7964186 at DNAH10 (dynein, axonemal, heavy chain 10). In conclusion, the authors were able to identify two regions of linkage influencing the development of male homosexual orientation: namely, 1) the pericentromeric region on chromosome 8 (maximum two-point LOD 4.08, maximum multipoint LOD 2.59), and 2) Xq28 (maximum two-point LOD 2.99, maximum multipoint LOD 2.76). This investigation was able to confirm genetic regions, which were implicated in previously published research, especially Xq28, including transcription factors, microRNAs, and various brain-expressed genes involved in neurodevelopment, neurotransmission, and functions and processes of the neuroendocrine system, as well as in social and affiliative communication, regulation of socio-sexual behaviors (like those odor-evoked) and emotional responses, mate selection, and mating success. Of note, the study was sufficiently statistically powered to detect moderate-to-major genetic *loci* contributing to the male homosexual trait.

The latter study (Mustanski et al., 2005) recruited a sample of mostly white (94.5%), college educated (87.4%), and of middle-to-upper socioeconomic status participants. The mean age for the gay siblings was 36.98 ± 8.64 years. In this study, male homosexuality was investigated by means of a structured interview that included a detailed questionnaire about the sexual history of the individual and the Kinsey scales of sexual attraction, fantasy, behavior, and self-identification. The study protocol had been advertised in local and national homophilic publications. The authors performed a genotyping analysis with 403 microsatellite markers at 10-cM intervals. Maximum LOD (mLOD) scores were computed separately for maternal, paternal, and combined transmission, to fully account for maternal loading of sexual orientation transmission and epigenetic factors acting on autosomal genes, using a linear model and assuming a multiplicative model. Nonparametric exclusion mapping of affected sib-pair data (ASP) analysis was carried out by utilizing ASPEX. The highest mLOD scores were computed at 3.45 at a position near D7S798 in 7qtel (∼7q35–q36) and at 1.96 near D8S505 in pericentromeric 8 (∼8p21–p11), respectively, with approximately equivalent maternal and paternal contributions in both cases. A maternal origin effect was found near marker D10S217 in 10qtel (∼10q26), with an mLOD score of 1.81 for maternal meioses and no paternal contribution. However, the authors could not find support for Xq28 linkage in the full sample. Given the previously reported evidence of linkage in this region, the investigators carried out supplemental research to clarify these contradictory findings. In the first follow-up, they re-analyzed their previously reported data and found an mLOD score of 6.47. When re-analyzing their current data, limiting the sample to those families previously reported, they were able to estimate an mLOD of 1.99.

Moreover, Ramagopalan et al. (2010) attempted to replicate Mustanski et al. (2005) findings of a putative linkage at 7q32, but they failed in detecting any statistical significance. They used the same Canadian cohort of 55 Caucasian families with two or more homosexual male siblings employed by Rice et al. (1999a). Genotype calls were obtained for 112 individuals and nonparametric linkage analyses were carried out using MERLIN. A LOD score peak of 2.86 could be obtained on chromosome 14 for SNP rs760335 (98.8 cM, position 9, 884,697; genome build 36). The adjacent SNP loci were rs733559 (96.8 cM; 92,809 308, LOD = 2.08) and rs742893 (99.7 cM; 94,222 866, LOD = 1.74). Performing modeling of marker–marker linkage disequilibrium in the dataset (r^2^ > 0.1) did not significantly modify the magnitude of LOD scores previously obtained (maximum LOD = 2.47). The empirical *p*-value for the linkage peak, computed by analyzing 1,000 simulated datasets of pedigree genotypes generated by MERLIN, was 0.256, showing proof of “failed to reach genome-wide significance”.

To overcome these contrasting results, recently, in 2021, Sanders and colleagues (Sanders et al., 2021a) conducted a meta-analytical study. They jointly analyzed the MGSOSO and the Hamer datasets, carrying out multipoint nonparametric linkage analyses with MERLIN on the two datasets initially separately because of different genotyping procedure (STRPs *versus* SNPs), then, finding the genetic positions of the respective markers in the Rutgers map and using nonparametric S-pairs and grid 1 cM options to perform multipoint linkage on both datasets, followed by combining LOD scores at each grid position across the marker sets. The authors as well incorporated a GWLS dataset on 55 families by using meta-analytic approaches on published summary statistics. The approach adopted by Sanders and colleagues has enabled to maximize the positional information from GWLSs of currently available family resources, helping prioritize findings from GWAS and studies leveraging other approaches. In conclusion, genetic components of human sexuality and, in particular, sexual orientation have been identified and highlighted, even though current understanding in terms of contributory genetic *loci* remains limited, consistent with the observation that genes involved in these highly complex traits may exert more modest effects than those detectable by linkage studies, warranting large-scale GWAS.

### Candidate gene studies of human sexual orientation

Candidate gene studies are studies that rely on the candidate gene approach to conduct genetic association studies focusing on associations between genetic variation within pre-specified genes of interest, and complex traits, phenotypes, or disease states. Candidate genes are selected for study based on *a priori* knowledge of the gene’s biological function and its impact on the trait or disease in question. We were able to identify only four candidate gene studies, carried out by DuPree et al. (2004), Macke et al. (1993), Yu et al. (2015), and Qin et al. (2018).

The former study (DuPree et al., 2004) failed in detecting the influence of the candidate gene CYP19 (aromatase cytochrome P450) on male sexual orientation. CYP19 is responsible for the conversion of androgens to estrogens and plays a key role in the sexual differentiation of the brain. The authors carried out linkage, association, and expression analyses by microarray analysis in a large sample of homosexual brothers using microsatellite markers in and around CYP19. No linkage could be detected, with a gene-specific relative risk of 1.5-fold being excluded at a LOD score of −2. Results of the transmission disequilibrium test (TDT) showed no preferential transmission of any of the CYP19 alleles in the sample under study. Expression analysis of aromatase mRNA could not compute any statistically significant differences between heterosexual and homosexual men.

The second study (Macke et al., 1993) formulated the hypothesis that DNA sequence variations affecting the androgen receptor gene can impact male sexual orientation, and tested the hypothesis by 1) measuring the degree of concordance of androgen receptor alleles in a sample of 36 pairs of homosexual brothers, 2) comparing the lengths of polyglutamine and polyglycine tracts in the amino-terminal domain of the androgen receptor in homosexual males and 213 unselected subjects, and 3) systematically screening the entire androgen receptor coding region for sequence variation by polymerase chain reaction (PCR) and denaturing gradient gel electrophoresis (DGGE) and/or single-strand conformation polymorphism analysis in a sample of 20 homosexual males with homosexual or bisexual brothers and one homosexual male with no homosexual brothers. Furthermore, the authors screened the amino-terminal domain of the receptor for sequence variation in an additional sample of 44 homosexual males, 37 of whom had one or more first- or second-degree male relatives who were either homosexual or bisexual. Based on the analyses’ findings, 1) homosexual brothers were as likely to be as discordant and concordant for androgen receptor alleles; 2) there were no large-scale differences between the distributions of polyglycine or polyglutamine tract lengths in the homosexual individuals *versus* the control group; and, 3) coding region sequence variation was not commonly found within the androgen receptor gene of homosexual individuals. Finally, the DGGE-based screening allowed the identification of two rare amino acid substitutions, namely, ser205-to-arg and glu793-to-asp, the biological significance of which is still not fully understood.

The third study (Yu et al., 2015) assessed the impact of the Val158Met genetic variation of the gene encoding catechol-O-methyltransferase (COMT). This gene is located on chromosome 22, has six exons, spans 27 kb, and codes for a protein of 271 amino acids, which plays a key role in regulating the embryonic levels of several catecholamine neurotransmitters (like dopamine, norepinephrine, and epinephrine) and estrogens. Since these hormones are involved in sexual behavior, also COMT has been hypothesized to be related to sexual orientation. The effects of the COMT gene SNP were assessed in a sample of 409 homosexual cases *versus* 387 heterosexual control Chinese men, by using a PCR-based assay and genotyping analysis. Statistically significant differences, both in genotype and alleles, between male homosexual individuals and controls could be found, suggesting a recessive model of the impact of the COMT gene SNP on male sexual orientation.

The latter study (Qin et al., 2018) was designed as a case-control study of 537 exclusively homosexual men and 583 exclusively heterosexual men, with data collected from March 2013 to August 2015. Data were analyzed using χ^2^ tests and logistic regression models, investigating the interplay of sociodemographic characteristics, childhood abuse experiences, and polymorphisms of COMT at rs4680, rs4818, and rs6267, and methylenetetrahydrofolate reductase (MTHFR) at rs1801133 on male homosexuality. The authors found a statistically significant association between a more frequent occurrence of physical (adjusted odds ratio [aOR] of 1.78), emotional (aOR of 2.07), and sexual (aOR of 2.53) abuse during childhood and male homosexuality. The polymorphisms of MTHFR at rs1801133 and COMT at rs4818 were as well associated with male homosexuality in the homozygote comparisons (T/T *versus* C/C at rs1801133, aOR of 1.68; G/G *versus* C/C at rs4818, aOR of 1.75). Finally, the authors could compute statistically significant interaction effects between childhood abuse experiences and the COMT and MTHFR genetic variants on male homosexuality.

### Genome-wide association studies of human sexual orientation

While, as expected most of candidate gene studies failed in finding a genetic association with a given gene and human sexual orientation, being relatively effective at identifying relative risk genes in Mendelian diseases, but inadequate when studying complex traits, subsequent GWAS found different putatively genetic factors involved in the inheritability of human sexuality. For instance, the 2013 GWAS investigation by Lawrance-Owen et al. (2013) was the first GWAS on human sexuality ever published, reflecting the shift from candidate gene studies and linkage studies, which look at specific genes and patterns of DNA sharing within families, respectively, to GWAS investigations. Whilst linkage analyses are the preferred strategy for identifying rare genetic variants with strong effects, GWAS represents the most powerful tool to identify common genetic variants with only small/tiny effects. Lawrance-Owen et al. (2013) GWAS was conducted by recruiting a sample of 979 healthy adults and identified variation upstream of SMOC1 (rs4902759, *p*-value of 1.41 × 10^−8^) as a genetic factor potentially mediating between prenatal sex hormones and digit ratio, which both have been extensively studied and implicated in homosexuality. The digit ratio is generally used as a biomarker for prenatal testosterone exposure, and, besides sexuality and sexual orientation, it has been correlated with a wide range of complex traits and disease conditions including prostate cancer, overweight and obesity, autism, and “Attention-Deficit/Hyperactivity Disorder” (ADHD). SMOC1 is a gene coding for the “SPARC Related Modular Calcium Binding 1” protein, which is instrumental in limb formation and development as well as in the sexually dimorphic development of the gonads and is mostly expressed in prostate tissues, in a sex hormone-dependent fashion. In a meta-analytical follow-up of this finding and an independent study, a probability of *p*-value of 1.5 × 10^−11^ could be computed, confirming the potential role of SMOC1 in shaping male sexuality.

The investigation by Sanders et al. (2017) succeeded in detecting a statistically significant association between the tag SNP for Sonic Hedgehog (SHH) rs9333613 polymorphism and male sexual orientation in a sample of 361 homosexual subjects and 319 Chinese male controls. SHH codes for a signaling molecule, which is key in fine-tuning embryonic morphogenesis and organogenesis, controlling the organization and development of the central nervous system, limbs, digits, and many other parts of the human body.

In 2017, Wang et al. (2012) conducted a GWAS of male sexual orientation on a primarily European ancestry sample of 1,077 homosexual men and 1,231 heterosexual men. The authors utilized Affymetrix single nucleotide polymorphism (SNP) arrays and were able to identify several SNPs with a *p*-value less than 10^–5^, in the 10^−5^–10^–7^ *p*-value range, as confirmed by a close visual inspection of the Manhattan plot. Each region with 9–10 SNPs exhibited *p* < 10^–5^ and various SNPs had 10^–7^<*p* < 10^–6^ and, with the most suggestive and “prominents” SNPs on chromosome 13 (minimum *p*-value of  7.5 × 10^−7^ for rs9547443) and chromosome 14 (*p*-value of 4.7 × 10^−7^ for rs1035144). The genes nearest to these peaks have biological functions plausibly relevant to the development of sexual orientation. On chromosome 13, the SNP is located between SLITRK6 (SLIT and NTRK like family member 6, ∼60 kb centromeric to region) and SLITRK5 (∼1.8 Mb telomeric), with SLITRK1 located ∼2.0 Mb centromeric, SLITRK6 is a gene coding for a protein, which is involved in some neurodevelopmental pathways and is mostly expressed in the diencephalon. This neuroanatomic structure contains a region that has been previously linked to male homosexuality (Bogaert and Skorska, 2020). SLITRK1 and SLITRK5 are other members of the SLITRK protein family, which are expressed at the brain level (especially, diencephalon and cerebral cortex), and are neuronal transmembrane proteins that are involved in a variety of biological functions, spanning from the regulation of neuronal outgrowth to survival, and synapse formation. On chromosome 14, TSHR is a gene coding for the thyroid-stimulating hormone receptor, and, besides the thyroid gland, it is also expressed in the brain, especially the hippocampus. TSHR-related genetic variants in intron 1 form a cluster of SNPs with association *p* < 10^–5^ and could explain, at least partially, previously published findings that have associated familial atypical thyroid function and male homosexuality. Furthermore, skewed X chromosome inactivation has been implicated in thyroid functioning and in Graves’ disease, as well as in mothers of homosexual men (Bogaert and Skorska, 2020). On pericentromeric chromosome 8 within a previously reported linkage peak, the authors were finally able to find support (*p*-value of 4.1 × 10^−3^) for an SNP association previously documented (rs77013977, *p*-value of 7.1 × 10^−8^), with the combined analysis employing a meta-analytic statistic that did not need direction of effect, namely, the Fisher’s combined probability test, and yielding a *p*-value of 6.7 × 10^−9^, which represents a genome-wide statistically significant association, reaching the threshold of genome-wide significance (i.e., 5 × 10^−8^). rs77013977 is an intronic SNP in NKAIN3, a gene codifying for the protein “Sodium/Potassium-Transporting ATPase Subunit Beta-1-Interacting Protein 3”, which potentially plays a key role for neuronal function. In conclusion, the authors found the strongest linkage support at pericentromeric chromosome 8, as well as at chromosomes 13, 14, and X, even though indirectly and more on a speculative basis. However, the study suffers from several limitations: among the most important shortcomings, the investigation mainly relies on two datasets, with relatively limited sample sizes. Also, it is exclusively focused on male homosexuals and mostly samples from European ancestry.

The study by Ganna et al. (2019), published in the prestigious journal “Science” in September 2019, represents the largest-ever GWAS of same-gender sexuality. The authors reported an analysis of 492,664 genomes. These included 477,522 genomes [408,995 from the United Kingdom (UK) Biobank and 68,527 from the United States (US) genetic testing company, 23andMe], which represented the discovery sample. This led to the identification of five SNP autosomal *loci* (located on five chromosomes: namely, 4, 7, 11, 12, and 15) significantly associated with same-gender sexual behavior (here, defined as ever-engaging in same-gender sexual contact). These *loci* were linked with the reproductive genital apparatus and the endocrine and olfactory systems. They withstood replication in independent samples consisting of 15,142 individuals from the United States and Sweden, meeting with stringent testing for individual statistical significance: overall, the authors were able to estimate that genetic influence accounted for 8%–25% of the variance in sexuality-related outcomes, even though the magnitude of measurable genetic influences was too small to yield reliable predictions of any individual’s same-gender sexual behavior based on their genome. Moreover, despite being large-scale, this study suffers from several shortcomings, including self-selection bias, as less than 6% and 2% of UK Biobank and 23andMe members, respectively, gave their consent to participate in the study. Also, the findings are not generalizable, since the authors, as *per* protocol, excluded subjects of non-European ancestry. Even if this is methodologically correct and sound, since GWAS should not be conducted on heterogeneous samples, this significantly constrains the degree to which the results of the study can be applied to other contexts and non-European populations. Moreover, of note, the birth years of the UK Biobank population ranged from 1937 to 1940 (from 40 to 69 years): homosexuality would have still been considered a mental disorder until 1973 when the American Psychological Association (APA) decided to remove it from its “Diagnostic and Statistical Manual of Mental Disorders” (DSM). In the UK, homosexuality would have been decriminalized only in 1967. Probably, the major shortcoming of the investigation is that, by operationalizing same-gender sexual behavior as ever-engaging in same-gender sexual contact, it conflates “constitutional/stable” and “facultative/unstable” same-gender sexuality, failing to capture the *continuum* spectrum of human sexuality (Hamer et al., 2021).

Finally, in 2021, the study by Hu et al. (2021) is a two-stage GWAS. In the first part of the investigation, the authors assessed a sample of 1,478 homosexual males and 3,313 heterosexual males in Han Chinese populations and were able to identify two genetic *loci* (rs17320865, Xq27.3, FMR1NB, P_meta_ of 8.36 × 10^−8^, OR of 1.29; rs7259428, 19q12, ZNF536, P_meta_ of 7.58 × 10^−8^, OR of 0.75) strongly and consistently associated with male sexual orientation. FMR1NB encodes the FMR1 Neighbor protein, which is mostly expressed at the levels of *spermatogonia*, spermatocytes, and spermatids, whilst ZNF536 encodes the Zinc Finger Protein 536, mainly expressed in the brain. In the second part of the investigation, the authors conducted a fixed-effect meta-analysis including individuals of Han Chinese (*n* = 4791) and European ancestries (*n* = 408,995). The meta-analytical follow-up was able to reveal three genome-wide significant *loci* of same-sex sexual behavior (rs9677294, 2p22.1, SLC8A1, P_meta_ of 1.95 × 10^−8^; rs2414487, 15q21.3, LOC145783, P_meta_ of 4.53 × 10^−9^; rs2106525, 7q31.1, MDFIC, P_meta_ of 6.24 × 10^−9^). Moreover, to provide biological insights into the identified genetic *loci*, the authors supplemented the GWAS investigation with a *post-mortem* study and an animal study. More in detail, they defined the average ZNF536-immunoreactivity (ZNF536-ir) concentration in the suprachiasmatic nucleus (SCN) as lower in homosexual individuals than in their heterosexual counterparts (0.011 ± 0.001 *versus* 0.021 ± 0.004, respectively, *p*-value of 0.013) in the *post-mortem* study. Also, the percentage of ZNF536 stained area in the SCN was significantly smaller in the homosexuals, compared with heterosexuals (0.075 ± 0.040 *versus* 0.137 ± 0.103, *p*-value of 0.043). Finally, more homosexual preference could be reported in a murine model (FMR1NB-knockout mice), along with statistically significant differences in the expression of serotonin, dopamine, and inflammation pathways that were reported to be related to sexual orientation when comparing CRISPR-mediated FMR1NB knockout mice to matched wild-type target C57 male mice.

### Linkage and genome-wide association studies of gender nonconformity/gender variance

Gender nonconformity, or more contemporarily gender variance, has been found to be one of the strongest correlates of homosexuality, displaying a high amount of familiarity. We were able to find only one linkage/GWL/GWAS investigation, carried out by Sanders et al. (Sanders AR. et al., 2021) in 2021. The authors assessed brothers in families with two or more homosexual brothers (409 concordant sibling pairs in 384 families, as well as their heterosexual brothers), who self-recalled their gender nonconformity. In order to map potentially contributory genetic *loci* for gender nonconformity, the authors carried out SNP genotyping analyses. The strongest linkage peaks, each with significant or suggestive two-point LOD scores and multipoint LOD score support, were on chromosomes 5q31 (maximum two-point LOD of 4.45), 6q12 (maximum two-point LOD of 3.64), 7q33 (maximum two-point LOD of 3.09), and 8q24 (maximum two-point LOD of 3.67). Of note, the latter did not overlap with the previously reported strongest linkage region for male sexual orientation on pericentromeric chromosome 8. Family-based association analyses were used to identify associated variants in the linkage regions, with a cluster of SNPs (minimum association *p*-value of 1.3 × 10^−8^) found at the 5q31 linkage peak. Genome-wide, clusters of multiple SNPs in the 10^–6^ to 10^–8^ *p*-value range were found at chromosomes 5p13, 5q31, 7q32, 8p22, and 10q23, highlighting glutamate-related genes and the potential role of the glutamatergic pathways on gender identity. However, this is the first reported GWL/GWAS investigation on gender nonconformity/gender variance and, as of today, the only study ever conducted. Further studies should be carried out to increase our genetic knowledge about gender nonconformity/gender variance and its relationships to male sexual orientation, to help advance our currently limited understanding of the biology of these associated traits.

## Discussion

Sexuality is a complex and multifaceted aspect of human experience that can encompass a wide range of factors, including biological, psychological, cultural, and social influences. Sexuality is an integral part of human identity and can play a significant role in relationships, self-expression, and overall wellbeing. According to previous research, we can cautiously conclude that there exists a substantial number of genes that impact human sexuality, a considerable proportion of which remain undiscovered. Recent advancements in human genome analysis have made it feasible to dissect the molecular basis of complex traits, such as sexual orientation, even if these traits are probably influenced by multiple genes and/or shaped by environmental or experiential factors, or, more probably, by some complex, non-linear combinations of these (McGuffin et al., 2001; Mustanski et al., 2002; Price, 2018; Richardson et al., 2019; McGuire et al., 2021). For instance, GWAS is a whole genome scanning method that simultaneously analyzes the relationship between thousands of SNPs across the entire genome and traits or diseases. This method does not require *a priori* hypothesis and can therefore detect any statistically significant genetic variation. However, while increasing the sample size may facilitate the identification of elusive genetic variations, this method still requires a significant investment of time and money, as well as addressing various statistical issues such as multiple comparisons, due to the need to analyze a large number of samples and data. It is worth noting, however, that predicting an individual’s sexuality accurately based on these SNPs is not possible since no single gene has a significant impact on sexuality.

According to McGuire (McGuire, 1995), genetic/genomic studies on human male homosexuality should fulfill the following criteria: 1) Reporting “valid and precise measures of individual differences”, 2) employing “appropriate methods to ascertain biological relationships”, 3) investigating “research subjects who have been randomly recruited”, 4) using “appropriate sample sizes”, 5) conducting “appropriate genetic models to interpret the data”, and 6) exercising “caution in interpreting biosocial effects from the observed phenotypic correlations.” At the time of the article (1995), McGuire concluded that “all studies of the genetic basis of sexual orientation of men and women have failed to meet one or more or any of the above criteria,” and, based on the present critical systematic review of the literature, we are afraid this conclusion still holds, even after decades of genomic research. A recently published computational simulation (Zietsch et al., 2021) on 358,426 individuals, incorporating genomic evidence from GWAS, showed that genetic effects associated with same-sex sexual behavior may, in individuals engaged in opposite-sex sexual behavior only, confer a mating advantage, reproductive, and survival benefit, explaining why genetic components of the homosexual trait have been evolutionarily maintained.

Some genetic *loci*, either X-linked or autosomal, have been reported - even though inconsistently and with a varying degree of replicability and reproducibility—to be associated with some human sexuality-related phenotypes and traits (Rodríguez-Larralde and Paradisi, 2009; Basavanhally et al., 2018; Chiang and Park, 2020). Autosomal *loci* have been explained by formulating various hypotheses, including overdominance, conferring male heterozygote advantages, whilst the “sexually antagonistic selection” theory (Camperio Ciani et al., 2015) could clarify the apparent paradox of X-linked *loci*, the molecular mechanisms through which persist and are transmitted “against all odds” of the evolutionary pressure.

It is of interest to note that linkage studies, even those leveraging whole genome scanning technique, are often methodologically inadequate and statistically underpowered to dissect genetic basis of complex traits, like sexual behaviors and sexuality-related phenotypes. Lander and Kruglyak (Lander and Kruglyak, 1995) have formulated clear guidelines for interpreting and reporting linkage results, including the need for external and independent replications of previously reported and published findings, besides having rigorous and stringent statistical thresholds to avoid a “flood of false positive claims” (Lander and Kruglyak, 1995; Lebrec et al., 2004).

Overall, the genetic/genomic analyses overviewed in the present systematic review seem to indicate that hundreds or thousands of genetic variants, each one with small but not negligible effects, can significantly contribute to the likelihood of describing themselves as a sexual minority or ever engaging in same-sex sexual behavior. Hence, these studies succeed more in capturing the overarching construct of human sexuality, in its broad meaning and scope, rather than dissecting the molecular basis of sexual orientation or same-sex sexual activity.

On the other hand, besides suggesting that human sexuality is polygenic, studies like those by Ganna et al. (2019) seem to suggest that most importantly, sexuality is a multidimensional construct. Rather than being monolithic, and one-size-fits-all, it is highly variable and incredibly diverse. Findings of modern GWAS, indeed, challenge the (usually unchallenged) assumption that sexuality should be conceived as a temporally rigid and stable *continuum*. From a methodological standpoint, the Kinsey scales may fail to capture sexual variability and heterogeneity, in terms of 1) sexual identity subtypes, 2) discrepancies between self-identification (self-declared sexual orientation) and behaviors (sexual activity), 3) longitudinal changes in sexual identity, 4) complexity, nuances, and fluidity of gender, 5) divergences among settings (with unexpected arousal patterns in laboratory *versus* real-life situations), and 6) the impact of interpersonal and situational (environmental, cultural, societal, spiritual/religious, political, historical, etc.) factors on shaping human sexuality.

For instance, the Kinsey scales have conflated distinct constructs, including the “degree of sexual attraction/behavior toward opposite-sex others and degree of sexual attraction/behavior toward same-sex others” (Zietsch and Sidari, 2020), operationalizing sexual orientation as a dimorphic trait categorized according to a “sexual binary logic”, with heterosexual and homosexual individuals representing two discrete, separate populations, at the opposite of the *continuum*. In other words, the two constructs of heterosexuality and homosexuality “are put in opposition to each other to yield a single number, such that a higher score on homosexual interest necessarily equates to a lower score on heterosexual interest” (Zietsch and Sidari, 2020). This “forced trade-off” (Zietsch and Sidari, 2020) has been, however, challenged by some experimental observations, including those by Jabbour et al. (2020), who were able to show that genital arousal to male and female *stimuli* are not associated in a statistically significant fashion, when controlling for genital arousal to neutral *stimuli* (and positively associated if the latter is not adjusted for). As such, the forced dichotomization operated by the Kinsey scales does not allow to appreciate the nuances of being bisexual/ambisexual. Moreover, the Kinsey scales do not incorporate or reflect the experiences of transsexual, intersex, transgender, and nonbinary individuals, whose lack of representation in the arena of sex research remains critical.

Ganna et al. (2019) add a further proof by investigating the genetic basis of both “ever” (*versus* “never”) engaging in same-gender sexual behavior and the ratio of same-gender to other-gender sexual contact (in other words, the varying proportion of same gender sexual partners). The authors showed a lack of genetic correlation between these two variable traits: 0.03 for men and −0.3 for women. The genetic correlation between the “exclusively same-gender” group and the “over two-thirds same-gender,” the “between one-third and two-third same-gender,” and the “less than one-third same-gender” groups was 0.95, 0.80, and 0.13, respectively. In other words, the genetic correlation between the “exclusively same-gender” group and its phenotypically closest and most distinct categories differed by seven times in magnitude, excluding from the “Kinsey *continuum*” several subtypes of sexual identity, like “mild bisexuality” and “mostly heterosexuality” (Diamond, 2021). Of note, the lessons we could learn from these results are manifold: 1) The knowledge concerning the “proportion of same-gender (*versus* other-gender) sexual behavior” does not allow to guess the intensity of “same-gender (*versus* other-gender) interest”; and 2) the knowledge about the “proportion of same-gender sexual behavior” does not allow to infer the “proportion of other-gender sexual behavior” (and similarly, the knowledge of the intensity of “same-gender interest” does not allow to guess the intensity of “other-gender interest”), since the “same-gender”- and “other-gender”-related parameters are not at the opposite ends of a single *continuum*, but seem to be orthogonal dimensions. Also, the findings of the study by Ganna et al. (2019) could potentially shed light on the molecular mechanisms underlying same-gender sexual behavior and the difference between an overarching, enduring sexual predisposition for one or both genders (a “state”) and a capacity/sensitivity for situational variability in sexual responsiveness (a “trait”). The latter construct is also known as “sexual fluidity” or “sexual flexibility” (Diamond, 2021; Katz-Wise and Todd, 2022). Ganna et al. (2019) showed that mostly heterosexual individuals or subjects sexually fluid/flexible are genetically different from exclusively heterosexuals and homosexuals. This could be due to the interaction between the trait of same-gender sexual interest and further traits like risk tolerance or openness to novelty (Diamond, 2021). From an epidemiological and clinical standpoint, individuals reporting both same-gender and other-gender behavior are more likely to suffer from greater mental and physical health issues than individuals who report exclusively same-gender or exclusively other-gender behavior. Moreover, from a temporal perspective, some studies have investigated stability and change in self-reported sexual orientation identity over time in youth, finding that up to 10% of males and 20% of females, at some point in their lives, described themselves as a sexual minority, whilst 2% of both males and females reported ever being “unsure” of their sexuality or being questioning their sexual orientation (Ott et al., 2011).

Findings reported by Ganna et al. (2019) were also sex-/gender-specific. The authors, indeed, found that the genetic correlation between men and women for “ever/never” was 0.65 for the UK Biobank sample and 0.34 for the 23andMe sample, both statistically significant, whilst the correlation between men and women was not significant for either the two samples (0.1 and −0.2, respectively). Looking specifically at the correlations between “ever/never” and “proportionality/ratio”, this was approximately null among men, and negative among women (−0.3 and −0.4, for the UK Biobank and the 23andMe samples, respectively). However, sex-/gender-specific aspects of human sexuality are overlooked. We found, indeed, that female sexuality is relatively understudied compared to male sexuality. The observation of some pedigree-based studies that male and female homosexuals exhibit different segregation patterns, with males usually having more gay brothers than gay sisters, while lesbians, on the contrary, having more gay sisters than gay brothers, seems to suggest that the factors responsible for this familial aggregation are, at least partially, distinct in men compared to women (Eckert et al., 1986; Jannini et al., 2010; Burri et al., 2011; Jannini et al., 2015; Bailey et al., 2016; Bogaert and Skorska, 2020; Diamond, 2021). Moreover, male sexuality tends to be bimodally distributed (heterosexuals *versus* homosexuals), whilst female sexuality exhibits a more continuous distribution with lower rates of homosexuality, and higher rates of bisexuality and “sexual openness” (Rausch et al., 2017; Diamond, 2021). In other words, women are more likely to report a bisexual than an exclusively same-sex sexual orientation/attraction, with men showing, instead, the opposite pattern. Men’s sexual orientations are closely linked to their pattern of sexual arousal to male *versus* female erotic stimuli, which is not necessarily true among women. Furthermore, female sexuality tends to less temporal stability with respect to male sexuality: women appear more likely than men to experience same-sex sexual attraction in the context of close affectionate relationships, and their patterns of sexual attraction/orientation appear more likely to exhibit change over time, even though some studies have disconfirmed this (Ott et al., 2011; Rausch et al., 2017). Of note, sex-/gender-specific aspects of human sexuality could also depend on the historically subordinate status of women, which “has made it more socially and economically risky for them to completely desist from heterosexual behavior, compared to men” (Diamond, 2021), as well as on the “hegemonic notions of rigid masculinity” (Diamond, 2021).

A further variability source of human sexuality is given by the interaction between the human and the surrounding environment. This could explain divergence between the correlations involving “ever/never” and those involving “proportionality/ratio” and an array of traits (risk behavior, mental health, personality, reproductive, and physical traits) in the study by Ganna et al. (2019). The interaction between genetic and environmental variables is relatively overlooked and understudied in the extant scholarly literature on the genetic/genomics basis of human sexuality. Indeed, we were able to find only one study—the investigation by Qin et al. (2018), dissecting the molecular basis of the impact of childhood abuse experiences on male sexual orientation. The paucity of studies in this regard can be explained taking into account the sensitive nature of these research foci, which may have societal, cultural, and political ramifications. Despite the well-established “role of learning and conditioning in human sexual response” (Diamond, 2021), this has been rarely and poorly “integrated into investigations of genetic and environmental influences on same-gender expression” (Diamond, 2021). This would be of high importance and would provide a broad, comprehensive picture of human sexuality. On the other hand, this notion and others similar can bring to mind the attempts to condition homosexuals and modify their sexual orientation and identity—the so-called “conversion” and “reparative” therapies, which have been proven as extremely harmful, besides being unsuccessful and not evidence-based.

Also, most of the research focused on the genetic aspects of same-sex sexuality tends to group all post-natal environmental factors together under the broad category of “environment.” However, it is important to consider childhood adversity as a distinct factor that should be approached differently. Not only is childhood physical/emotional/sexual abuse from 1.2 to 3.8 times as common among individuals engaging in same-sex behavior (Friedman et al., 2011), but this type of childhood experience is known to interact with a number of polymorphisms (including monoamine oxidase A, MAO-A, dopamine receptors D_2_ and D_4_, DRD2 and DRD4, dopamine transporter, DAT1, serotonin transporter, 5-HTTLPR, brain-derived neurotrophic factor, BDNF, FK506 binding protein 5, FKBP5, and COMT), to shape trait expression and moderate the detrimental impacts of postnatal family adversity on child externalizing behaviors, such as aggression and conduct disorder (Weeland et al., 2015), as well as suicide and suicidal behaviors (Berent et al., 2020), depression, other affective disorders (Li et al., 2020), and post-trauma stress disorder (PTSD) (Jin et al., 2019), among others. Taken altogether, it seems logical to consider childhood adversity as a separate factor because it is an experience that influences the developing nervous system to varying levels of nurturance and threat in adulthood. Therefore, it can be regarded as a special type of environmental cue that carries significant importance (for instance, in accordance with Del Giudice et al. (2011) adaptive calibration model of stress responsivity, and Ellis et al. (2022) integrated model of threat-based forms of harshness, deprivation-based forms of harshness, and environmental unpredictability).

Only a small number of genetic studies have taken into account the moderating effects of childhood adversity, and in some cases, its potential epigenetic effects. This constitutes a significant flaw. One of the reasons why sexual orientation researchers often overlook childhood adversity is their reluctance to provide any support to anti-gay activists who argue that sexual abuse is the “cause” of same-sex sexuality. It is crucial to note that there is no evidence to support such a claim. However, there is reliable data indicating that childhood adversity, encompassing various forms beyond physical, sexual, and emotional abuse, including poor parent/child relationships, maternal separation, child neglect, family conflict, social isolation, low socioeconomic status, or even extreme poverty, and institutional rearing, among others, communicates to the developing nervous system that life is likely to be brief, harsh, and unpredictable. Studies suggest that such experiences have the capacity to activate or deactivate different genes as well as pathways linked to the human immune system (proinflammatory cytokine production and release, immune dysregulation, and impaired immunologic response to tumors and infectious agents) and the brain (involving the synaptic transmission within brain regions and neural circuitries that mediate sensory cue processing and behavioral regulation; stress resilience within stress-sensitive brain regions like the amygdala, the hippocampus, and the hypothalamus, as a major part of the hypothalamic-pituitary-adrenal axis, the human key stress response system; synaptic plasticity within the prefrontal cortex; aminergic pathways and, in particular, dopaminergic signaling within the *nucleus accumbens*, and other neurobiological mechanisms and neuroregulatory networks) (Gillespie et al., 2019; Dieckmann et al., 2020; Chen et al., 2021; Czamara et al., 2021; Schuler et al., 2022; Sumner et al., 2022; de Carvalho et al., 2023; Dunn et al., 2023). Childhood and, more generally speaking, early-life adversity represent potent environmental exposures, which become “biologically embedded and contribute to adverse mental and physical health” (Sumner et al., 2022). These exposures interact with the children’s genetic make-up (in terms of genetic polymorphisms and other forms of variation), which in turn results in a range of structural and functional biological changes and behavioral adaptations across several levels and multiple pathways (Berens et al., 2017). These changes, in turn, impact adult mental and physical outcomes (Nelson et al., 2020), even though the precise mechanisms have to be elucidated yet. Given their importance, all these issues should warrant much greater attention from the scientific community.

### Future prospects

As interestingly noted by Vázquez (2022), published reports have utilized various operationalized definitions of sexuality, often in an inconsistent way. The author has identified four major markers or groups/clusters of markers: 1) Self-identification, attraction, fantasy, and behaviour (mostly employed by Hamer et al. (1993) (Hu et al., 1995); 2) self-identification, corroboration from secondary sources, and stereotypes (used by Rice et al. (1999a)); 3) self-identification and sexual feelings (utilized by Sanders et al. (2015)); and 4) sexual behavior (employed by Ganna et al. (2019)). These differences in phenotyping make it difficult to compare and contrast the various findings. Moreover, according to González Vázquez (Vázquez, 2022), each one of these “notions” is unsatisfactory, lacking predictive and explanatory power.

A consensus on what is meant by “human sexuality” should be reached, also engaging the 2SLGBTQIAP + community, as recommended by several institutions, such as the “International Gender Diversity Genomics Consortium”. Of note, only a few studies applied participatory research and public involvement approaches. Sanders et al. (2015) liaised with 2SLGBTQIAP + groups, whilst Ganna et al. 2019) communicated their study findings to the broader public, by organizing workshops and seminars in which representatives of the public, 2SLGBTQIAP + activists, and scholars discussed the scientific rationale, and the results, as well as the political and social implications of the study.

Adopting such approaches is paramount, especially when dealing with “sensitive” topics like human sexuality, that has important implications in terms of public understanding of science and public trust towards science, as well as impact on decision- and policymakers, given that, as of December 2020, 69 United Nations (UN) member States still continue to criminalize consensual same-sex sexual activity.

A more nuanced and “consilient” approach (to quote Wilson, the father of sociobiology) should be adopted when dealing with human sexuality, carefully (re-) thinking of the epistemological, ethical, and philosophical basis and socio-cultural and anthropological implications of behavioral genomics (Savulescu et al., 2021; Hammack-Aviran et al., 2022).

In conclusion, even though an accumulating body of scholarly evidence seems to highlight genetic contributions to human sexual orientation, especially in males and in European/North American populations, our current understanding of contributory *loci* is still limited, probably consistent with the complexity of the trait. Further increasing genetic knowledge about human sexual orientation, especially *via* large-scale GWAS studies and in female and non-European/non-North American populations, could help advance our understanding of the biology of this important trait, really and fully embracing its pluralism, and diversity, potentially providing new biological insights into the genetic basis of human sexuality from a wider population scope. To achieve this ambitious goal, *consortia* of cooperating multidisciplinary/interdisciplinary research teams will be necessary to collect sufficiently large samples and have adequate statistical power to detect also tiny effects of putative sexuality/sexual orientation-related genes, along with exploring new research areas and *foci* such as behavioral epigenetics/epigenomics, given the documented parent-of-origin effects, especially in male homosexuality.

## Data Availability

The original contributions presented in the study are included in the article/supplementary material, further inquiries can be directed to the corresponding author.
